# Complement C1s as a diagnostic marker and therapeutic target: Progress and propective

**DOI:** 10.3389/fimmu.2022.1015128

**Published:** 2022-10-06

**Authors:** Jun Ye, Peng Yang, Yili Yang, Sheng Xia

**Affiliations:** ^1^ Department of Immunology, School of Medicine, Jiangsu University, Zhenjiang, China; ^2^ Center for Translational Medicine, The Affiliated Taizhou People’s Hospital of Nanjing Medical University, Taizhou, China; ^3^ Department of Emergency Medicine, The First Affiliated Hospital of Soochow University, Suzhou, China; ^4^ China Regional Research Centre, International Centre of Genetic Engineering and Biotechnology, Taizhou, China

**Keywords:** complement, C1s, biomarker, protein, immunity

## Abstract

The molecules of the complement system connect the effectors of innate and adaptive immunity and play critical roles in maintaining homeostasis. Among them, the C1 complex, composed of C1q, C1r, and C1s (C1qr_2_s_2_), is the initiator of the classical complement activation pathway. While deficiency of C1s is associated with early-onset systemic lupus erythematosus and increased susceptibility to bacteria infections, the gain-of- function variants of C1r and C1s may lead to periodontal Ehlers Danlos syndrome. As C1s is activated under various pathological conditions and associated with inflammation, autoimmunity, and cancer development, it is becoming an informative biomarker for the diagnosis and treatment of a variety of diseases. Thus, more sensitive and convenient methods for assessing the level as well as activity of C1s in clinic samples are highly desirable. Meanwhile, a number of small molecules, peptides, and monoclonal antibodies targeting C1s have been developed. Some of them are being evaluated in clinical trials and one of the antibodies has been approved by US FDA for the treatment of cold agglutinin disease, an autoimmune hemolytic anemia. In this review, we will summarize the biological properties of C1s, its association with development and diagnosis of diseases, and recent progress in developing drugs targeting C1s. These progress illustrate that the C1s molecule is an effective biomarker and promising drug target.

## Introduction

The complement system consists of more than 30 proteins found in soluble form or attached to cell membranes. Their biological functions include cell lysis, opsonization, degranulation of mast cells and basophils, activation of B lymphocytes, and clearance of immune complex and apoptotic cells. With its components mostly existing as precursor zymogens in the plasma, the system can be activated through the classical pathway (CP), alternative pathway (AP), and the lectin pathway (LP). Of note, the CP is typically activated by the immune complex formed by specific antibodies and antigens such as microbes, allo- and auto-antigens. Through the activation, the antibodies promote the formation of membrane attack complex that often lyses target cell directly, generate complement component-mediated opsonization, and augment inflammatory response. Thus, the classical pathway activation of the complement is involved in the initiation, progression, and prognosis of many bacterial and viral infections-induced responses, autoimmune diseases, and cancers (1–5). Conversely, inherited deficiency and hypofunction of the complement system are associated with primary immunodeficiencies as well as systematic lupus erythematosis and hereditary angioedema (6–8).

**Figure 1:**
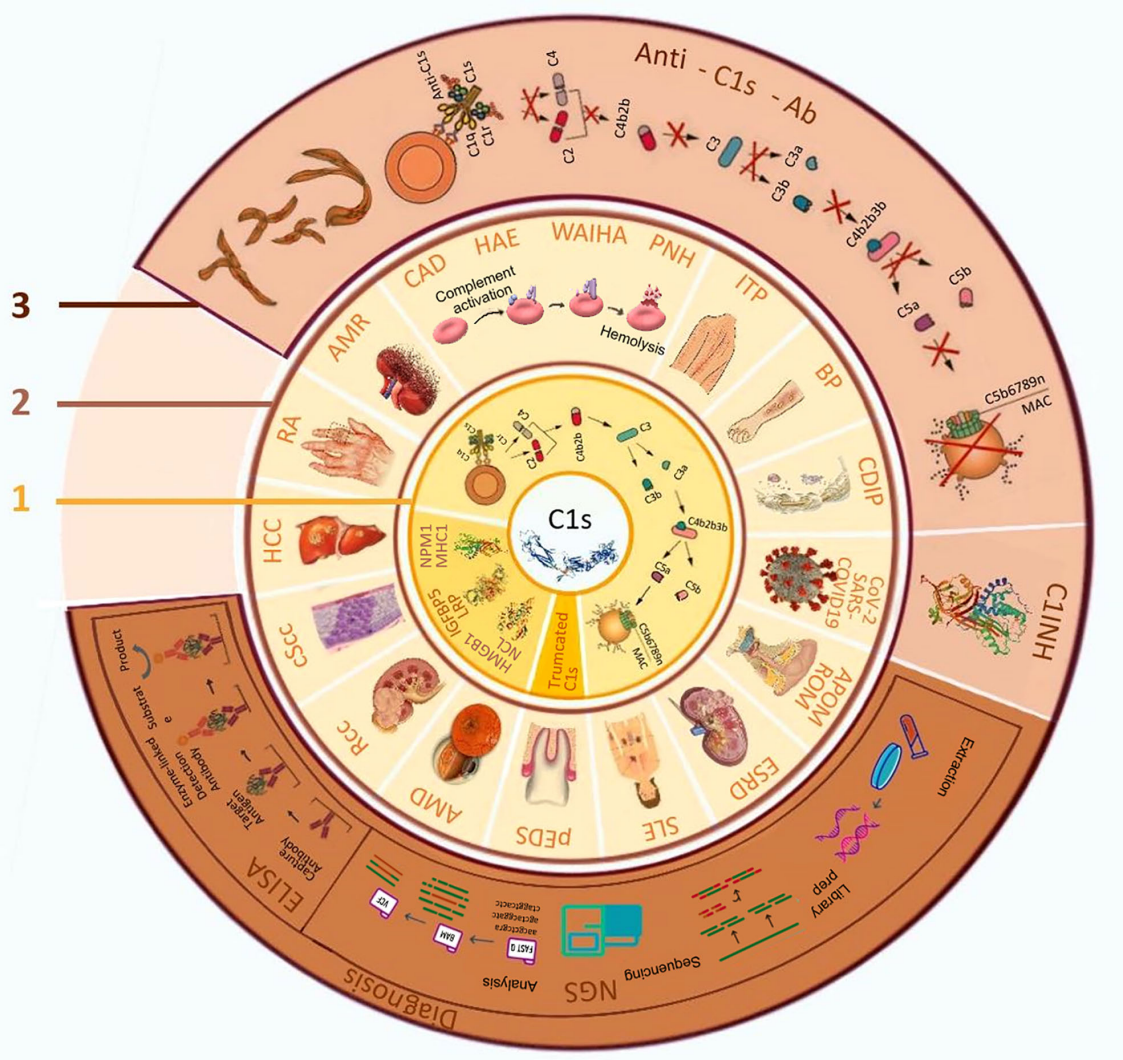
The biological function of C1s, related diseases and its application in diagnosis and treatment. 1. Biological functions of C1s; 2. C1s related diseases; 3. Application of C1s as a target in disease diagnosis and treatment. AMD: age-related macular degeneration; AMR: antibody-mediated rejection; APOM: acute pneumococcal otitis media; BP: bullous pemphigoid; CAD: cold agglutinin disease; CDIP: chronic inflammatory demyelinating polyradiculoneuropathy; COVID-19, coronavirus disease 2019; cScc: cutaneous squamous cell carcinoma cells; C1INH: C1 esterase inhibitors; ELISA, enzyme-linked immunosorbent assay; ESRD: end-stage renal disease; SLE: systemic lupus erythematosus; HAE: hereditary angioedema; HCC: hepatocellular carcinoma; HMGB1:high-mobility group box 1; IGFBP5: insulin-like growth factor binding protein-5; ITP: idiopathic thrombocytopenic purpura; LRP: low-density lipoprotein receptor-related protein; MAC: membrane attacking complex; MHC1: Major histocompatibility complex 1; NCL: nucleolin; NGS, next-generation sequencing; NPM1: nucleophosmin 1; pEDS: periodontal ehlers-danlos syndrome; PNH: paroxysmal nocturnal haemoglobinuria; RA: rheumatoid arthritis; Rcc: renal cell carcinoma; ROM: recurrent otitis media; SARS-CoV-2, severe acute respiratory syndrome coronavirus 2; WAIHA: warm autoimmune hemolytic anemia.

At molecular level, C1 is a complex composed of one C1q, two C1r, and two C1s subunits (C1qr_2_s_2_). In the classical pathway, antibodies complexed with antigens bind C1q and change its conformation, leading to activation of the protease activity of C1r, which in turn cleaves and activates C1s. C1q can also be activated through binding to C-reactive protein and the surface of pathogens (9, 10). The activated C1s subsequently cleaves substrates C4 and C2, resulting in the formation of C3-convertase (a complex of C4b and C2b) that splits C3 into C3a and C3b, which then cleaves C5 and triggers the formation of so-called membrane attack complex (MAC) consisting of C5b, C6, C7, C8, and polymeric C9 (**Figure 1-1**). Thus, monitoring C1s activity and targeting C1s with small molecular inhibitors and monoclonal antibodies have been the focus of many studies in recent years (11–14).

The *C1s* gene, located on the short arm of chromosome 12 (12p13.31), contains 12 exons and encodes a precursor C1s protein of 688 amino acids (15, 16). At its N-terminal region, the protein harbors CUB2 and CUB1 domains connected through an epidermal growth factor (EGF)-like domain, which are followed by complement control protein (CCP) modules CCP1 and CCP2. The C-terminal region of C1s has a serine protease (SP) domain (residues 423-688) that contains an activation peptide-like fragment (residues 423-437). During its activation, C1s is cleaved into a heavy chain of 422 amino acids (A-chain) and a light chain of 251 amino acids (B-chain). Of note, C1s is glycosylated at a number of sites and its serine protease activity shows trypsin-like specificity and cleaves arginyl bonds in substrate proteins.

Interestingly, besides C4 and C2, activated C1s also acts on many cellular proteins and exerts multiple regulatory actions. It has been found that activated C1s cleaves major histocompatibility complex I (MHC I) from the cell surface and hydrolyzes 2 microglobin, affecting T cell-mediated immune response (17, 18). It also cleaves insulin-like growth factor binding protein 5 (IGFBP5) from cultured fibroblast, whereas C1q activates the Wnt signaling pathway (17–20), which may affect cell growth as well as neuronal connectivity (21, 22). In addition, low-density lipoprotein receptor-related protein (LRP6), nucleophosmin 1 (NPM1) and nucleolin (NCL) are all substrates of C1s (20, 23). Interestingly, C1s can cleave high-mobility group box 1 (HMGB1) protein, a notable auto-antigen in autoimmune diseases (24). Although these proteolytic activities are far less efficient than the canonical C4 and C2 cleavages leading to complement activation, it is conceivable that they take part in tissue renewal process that reduces the immunogenicity of tissue debris and decreases the likelihood of autoimmunity induced by auto-antigens and danger associated molecular patterns (DAMPs). Thus, C1 activation affects physiological and pathological processes through multiple mechanisms (**Figure 1-1**).

## C1s and human diseases

The critical roles of the complement system and C1s in maintaining homeostasis make their dysfunction associating with a variety of disease. While mutation of C1s is associated with rare genetic diseases and susceptibility to infections and autoimmune disorders, ongoing studies have indicated that the aberrant activation of C1s contributes to the development of autoimmune and infectious diseases and cancer, and serves as an informative biomarker and therapeutic target (**Figure 1-2**).

## Loss and gain function mutation of C1s

It has been shown that deficiency of the classical pathway proteins of the complement system is strongly associated with the development of early-onset systemic lupus erythematosus (SLE), an autoimmune disease characterized by auto-antibody against multiple tissues and organs (3). The likely mechanisms include the lack of activated proteases, such as C1r and C1s, to digest the potential auto-antigens form dying cells and immune complexes and lack of modulation of the immune response set-point by activated complement components. Of note, studies in mouse model indicated that C1r/C1s deficiency alone is insufficient in inducing SLE (25), suggesting that the development of the disease is affected by multiple factors. Further analyses found that the appearance of stop codon (Y204X) in exon 6 that leads to premature protein translation termination is a commonly identified C1s mutation in SLE patients (26). It has also been reported in a family study that the propositus contains homozygous nonsense mutation R534X, and both parents and 4 family members in 2 generations are heterozygous of the mutation (27). Interestingly, it was found in an extended Japanese family that C1s deficiency may result from heterozygous combination of different mutations, including a 4-bp TTTG deletion in exon 10 that results in a frame shift mutation (28), a stop codon mutation (E597X) in exon 12 that encodes a C1s missing 80 amino acids at the C-terminal (29), and a missense mutation G630Q (30).

Periodontal Ehlers Danlos syndrome (pEDS) is a rare disorder characterized mostly by early-onset periodontitis. It has been shown that this subtype of Ehlers Danlos syndrome is caused by mutations in the genes of C1r and C1s (31). The mutations include heterozygous missense, in frame insertion, and deletion in C1r (15 families) or C1s (2 families). A recent study also identified multiple heterozygous C1s mutations (G962C, T961G, and T961A in an extended family as pathogenic variants (32). Interestingly, it was shown that the mutated C1s Val316del and Cys294Arg were produced in the cells as truncated proteins that lost their N-terminal domain (33). It is evident that these mutations led to constitutive activation of C1r and C1s and the development of pEDS. Structural analyses showed that C1r and C1s interact *via* an extensive interface encompassing the N-terminal regions of both proteins (34). Therefore, these mutations might disrupt the binding between C1r and C1s and facilitate aberrant C1s activation (**Figure 1-1**). It is also conceivable that the mutation break the interaction of C1r-C1s and C1q, uncoupling C1s activation from C1 complex binding.

## Autoimmune diseases

As an important system involved both in innate and adaptive immunity, the aberrant activation of the complement is present in many autoimmune diseases, including cold agglutinin disease (CAD), rheumatoid arthritis (RA), and systemic lupus erythematosus (SLE) (13, 35). CAD is a rare autoimmune hemolytic anemia due to the production of IgM auto-antibody (cold agglutinin) against surface antigens of red blood cells (RBCs). The auto-antibodies bound to RBCs activate the complement CP, leading to formation of MAC and hemolysis that manifested as anemia, severe hemolytic crisis, and even death (36, 37). The success of anti-C1s mAbs in the treatment of CAD further demonstrated the critical role of C1 activation in the pathogenesis (38). Additionally, neuromyelitis optica spectrum disorder, characterized by central nervous system inflammation and demyelination, is mediated by anti-aquaporin-4 (anti-AQP4) auto-antibodies targeting astrocytes and subsequent complement activation (39). Clinical studies revealed that anti-C5 antibody eculizumab effectively prevented the disease development (40). It is interesting to further determine whether blocking C1 activation can also alleviate the tissue damage and the autoimmune disease.

Rheumatoid arthritis (RA) is an autoimmune disease of the joints, which results in inflammation and thickening of the joint capsule, and loss of underlying cartilage and bone. C1s activation has been found in the degenerative osteoarthrotic cartilage, but not in normal articular cartilage. The activated C1s was mostly restricted to the severely degraded part of cartilage in osteoarthritis, indicating the participation of C1s in the inflammation and the destroy of cartilage and bone (41). It has also been found that tumour necrosis factor alpha (TNF-α) increased the production of C1s in cultured chondrocytes, suggesting that the inflammatory and destructive roles of C1s may be amplified in the inflammatory joint.

Paradoxically, the roles of C1s in SLE is complicated. While C1r and C1s deficiency predisposes to lupus (42–44), C1s levels in plasma from SLE patients are significantly higher than that in normal subjects, likely reflecting that the increased auto-antibodies activate C1 (45). It has been shown that auto-antibodies against C1s were detected in 7 out of 15 patients with SLE, and may activate C1 and enhance C4 and C2 cleavage (46). However, the positive rate of anti-C1s auto-antibody was markedly lower (6.9%) in a study of a larger group of patients with lupus nephritis, and the presence of anti-C1s and anti-C1r auto-antibodies did not correlate with the clinical parameters of the disease (47). It is worth noting that C1q is frequently targeted by auto-antibodies (anti-C1q), which correlates best with active renal disease in SLE patients (48). It is interesting to further explore how the anti-C1q auto-antibodies affect the complement system and the disease progress. Further,the auto-antibody against C1q could be induced by antigen-derived from Epstein-Barr virus, a well-known trigger of SLE (49). Thus, there are complicated interactions between C1 activation and the development and progress of SLE. It is worth to further explore whether the C1 components may be utilized as diagnostic markers for disease initiation and progress, and as targets of therapeutic intervention at different stages of the disease.

## Cancer

Given the close association of complement with immunity, it is not unexpected that activation of C1 is associated with the development, progression, metastasis, and treatment of a variety of cancers (50, 51). In clear cell renal cell carcinoma, local production and activation of C1s drove tumor progression and was associated with poor prognosis (52). Further explorations suggested that C1s facilitates the cancer progression by triggering complement activation, and by modulating the tumor cell phenotype and tumor microenvironment in a complement cascade independent manner (53). One of the cascade independent function may be mediated by the C1 receptor on monocytes, whose engagement drives the cells to migrate into tissues, differentiate into macrophages or dendritic cells (DCs), and initiate adaptive immunity (54). It has also been found that macrophage produced C1q and tumor cell-derived C1r, C1s were assembled in clear cell renal cell carcinoma and resulted in an immunosuppressive microenvironment that promotes tumor progress (55). Therefore, C1s and CP of the complement system can act as an effector of anti-tumor immunity as well as an promoter of the tumorigenic microenvironment in tumor.

## Infectious diseases

It is well-known that complement can be activated through CP, LP, and AP on the surface of microbes, leading to the generation of MAC that kills bacteria and parasites *via* forming transmembrane pores (56). The complement may also mark bacteria for phagocytosis and processing by antigen-presenting cells to stimulate adaptive immune cells. While bacteria may utilize a variety of strategies to evade the action of complement, deficiency in the complement system results in susceptibility to infections of various microbes, especially encapsulated bacteria (57). Therefore, activation of complement is a hallmark of bacterial infection. For example, it has been reported that the level of C1s was increased in children with acute pneumococcal otitis media (58). Further, the elevated complement complexes detected in the sera of children with recurrent otitis media contained C1r and C1s, and were able to kill the bacteria (58). Complement activation is also involved in virus infection. Of note, it was shown that up-regulation of C1s led to host cell damage *via* the classical pathway in a model of SARS-CoV-2 infection (59). Clinical investigation found that the mean 50% hemolytic complement (CH50) level in COVID-19 patients was significantly lower compared to that in healthy individuals, likely due to sustained CP activation in these SARS-CoV-2 infected patients (60). Further studies found that antibody-mediated increase of CP activation was highly associated to COVID-19 disease severity (61), although the downstream effects of this activation may differ depending the disease status of the individual and on the specific antigen targeted (62). Interestingly, the C1 esterase inhibitor (C1INH), which inhibits C1r and C1s activation, significantly reduced fever and inflammation in patients with COVID-19 (63). Due to the large inhibiting spectrum of C1INH, these findings suggest, but did not prove that activated C1s is an effector, biomarker, and potential therapeutic target of various infectious diseases.

## C1s as a therapeutic target

The critical role of the complement system in innate and acquired immunity and various diseases prompted various efforts to develop specific therapeutic agents (**Figure 1-3**). C1s is an attractive target since its inhibition blocks the system at an early stage of the complement cascade. While peptide that binds C1q and blocks C1r and C1s activation have been developed (64), finding selective small molecular inhibitors for the serine protease is challenging. A number of reported molecules did not exhibit high specificity and adequate pharmacokinetics yet (65). A large machine learning-based virtual screening was carried out and found a series of potential valuable inhibitors (66). Further combination of *in silico* and *in vitro* approaches identified hit compounds with new chemo-types and high potency in inhibiting C1s (67). It is interesting to determine whether these inhibitors can interfere the activity of C1s under physiological and pathological conditions.

C1INH is a natural plasma protein whose level is further increased upon stress and inflammation. It has a characteristic serpin domain and irreversibly binds to and inactivates C1r and C1s proteases (68). Of note, C1INH also prevents complement activation through the lectin pathway and exhibits inhibition on multiple proteases, notably kallikrein, fXIa, and fXIIa (69), and therefore modulating the kinin, coagulation, and fibrinolytic systems. It has been proposed that the protein may be utilized in a variety of diseases, including septic shock, reperfusion injury, hyperacute transplant rejection, traumatic and hemorrhagic shock. Currently, C1INH prepared from human plasma (Cinryze™) has been approved for the treatment of hereditary angioedema, a rare autosomal dominant disease manifested by recurrent acute attacks of edema that results from C1INH deficiency (70).

Monoclonal and engineered antibodies have been the most advanced agents for blocking complement activation, including antibodies against C1s (**Table 1**) and C5 (79). In particular, anti-C1s antibody sutimlimab significantly inhibited Ig-induced activation of B cells derived from patients with rheumatoid arthritis, indicating that targeting C1s may not only block complement-mediated tissue damage, but also suppress the activation of autoimmune B cells, which is a critical pathogenic factor in many autoimmune diseases (13). For example, bullous pemphigoid (BP) is a potentially life-threatening skin disease characterized by blister formation resulted from auto-antibody-mediated complement action and subsequently inflammation and tissue damage. In an *ex vivo* human skin cryosection assay, anti-C1s antibody TNT003 effectively prevented complement activation induced by the bullous pemphigoid auto-antibodies (80, 81). In patients with thrombocytopenic purpura, auto-antibody against platelet membrane protein leads to activation of complement and destruction and reduction of platelet. Sutimlimab inhibited the CP of complement activation and significantly decreased plasma deposition of C3b and C5b-9 in these patients. Meanwhile, the direct damaging and reticuloendothelial system clearance of platelets were markedly reduced (14). Similar studies found that anti-C1s antibodies were effective in blocking complement activation induced by auto-antibodies from patients with several types of autoimmune hemolytic anemia, such as paroxysmal nocturnal hemoglobinuria and CAD (14).

**Table 1:** Registered clinic trials with C1s targeted mAbs.

Disease	Medicine	Clinical staging	NCT NO
Bullous Pemphigoid Cold Agglutinin Disease Warm Antibody Type Autoimmune Hemolytic Anemia End stage renal disease	BIV009	1	NCT02502903 (71)
Cold Agglutinin Disease	sutimlimab (BIVV009)	3	NCT03347422 (72)
Cold Agglutinin Disease	Sutimlimab	3	NCT03347396 (73)
Idiopathic Thrombocytopenic Purpura	sutimlimab (BIVV009)	1	NCT03275454 (74)
Idiopathic Thrombocytopenic Purpura	BIVV020	2	NCT04669600 (75)
Autoimmune hemolytic anemia	BIVV020	1	NCT04802057 (76)
Autoimmune hemolytic anemia	BIVV020	1	NCT04269551 (77)
Chronic Inflammatory Demyelinating Polyradiculoneuropathy	BIVV020	2	NCT04658472 (78)

Antibody-mediated rejection (AMR) in solid organ transplantation is caused by donor-specific antibodies. It is characterized by the activation of the complement system and infiltration of macrophages (CD4^+^ and CD68^+^). In a study with aortic endothelial cells, it was demonstrated that the activation of the complement by anti-MHC antibodies facilitated the recruitment of monocytes. The anti-C1s antibodies TNT003 and TNT009 inhibited the antibody-induced complement activation and blocked C3d deposition (82), which are critical for the microvascular inflammation during AMR (83). The inhibitory effects of these antibodies on CP activation have also been demonstrated in patients (84). Whether they can reduce the microvascular inflammation during AMR and prolong the survival of transplants remain to be further explored. Interestingly, it was found that C1s-specific monoclonal antibody TNT005 did not abolish the therapeutic effects of anti-*Neisseria meningitidis* and *Streptococcus pneumoniae* antibodies, whereas simultaneous inhibition of CP and AP blocked the killing function of the anti-bacterial antibodies (85). Thus, targeting C1s may specifically prevent CP activation without abating the alternative and lectin pathways of complement activation and their associated immunological effector functions, and might also reduce the production of auto-antibodies.

There have been a number of registered clinical trials that target C1s with mAbs for the treatment of hereditary angioedema (HAE), chronic inflammatory demyelinating polyradiculoneuropathy (CIDP), cold agglutinin disease (CAD), thrombocytopenic purpura, and autoimmune hemolysis (**Table 1**). Sutimlimab (also known as TNT003, BIVV-009) is an humanized IgG4 mAb against C1s. It was reported that 7 of 10 CAD patients treated with sutimlimab achieved remission, and all 6 patients with a history of blood transfusion became transfusion-free during treatment (11). In a named patient program, 7 CAD patients responded to re-treatment, and sutimlimab increased hemoglobin from a median initial level of 7.7 g/dL to a median peak of 12.5 g/dL (*P* = 0.016). All patients remained transfusion free while receiving sutimlimab, and there were no treatment-related serious adverse events. In another trial with sutimlimab, 13 out of 24 (54%) CAD patients reached the primary end point. Of the 11 patients that did not meet the predefined criteria, 6 of them showed a therapeutic response (12). These and further clinical trials led the recent approval of sutimlimab for the treatment of CAD by the US FDA (86). Of note, another anti-C1s antibody BIVV020, whose tolerability safety study has been completed in patients with CAD, is being actively tried in clinic for the treatment of CAD, immune thrombocytopenia, and antibody-medediated transplant rejection (**Table 1**). It is conceivable that these antibodies may be proved as an effective therapeutics for additional forms of autoimmune hemolytic anemia in not-so-distant future.

## C1s determination in biological samples

Various methods have been developed to detect the complement in clinical samples.Traditionally, the total complement activity of CP is quantified by hemolysis assay in serum, based on the dilution achieving 50% of hemolysis (CH50). Functional ELISAs using coated IgM and colorimetric substrate have also been developed to detect the activity of CP in serum (87, 88). However, these assays only evaluate the overall CP complement activity, but not the levels of individual complement components. The C1 assay through capture of C1q in serum may represent a better measurement of C1s (89). It has also been shown that the analyses of CP components, such as C1q and C1r could be achieved quantitatively or semi-quantitatively by immunodiffusion, ELISA, and nephelometry assays (90). However, these methods only detect the immuno- reactivities of target complement components, and do not necessarily reflect their activity states. For the studies of congenital complement deficiency or for the study of genetic polymorphisms in populations, next-generation sequencing (NGS) and qPCR are often deployed to analyze particular components (91). Therefore, there are no validated C1s quantification assays available in clinical practice currently, and the quantification of C1s in research is also difficult due to limited reliable antibodies and lack of standardized assays.

The level of the total C1s protein can be detected by bilateral diffusion (92), ELISA (27), gelatin zymography (45), and LC-MS/MS (93) (**Table 2**). As the protein is normally present in blood and tissues, it is important and highly desirable to assess activated C1s to understand its exact roles under physiological and pathological conditions. It has been shown that the activity of C1s can be measured by the cleavage C2 and C4 (94). However, the assay is only semi-quantitative, and C2 and C4 can also be cleaved by other serum proteases such as MASP2. Interestingly, antibodies recognized activated C1s have been reported, which make it possible to determine the protein through western blot. It has been shown that such antibodies detected activated C1s in degenerative cartilage matrix of RA through immunohistochemistry (96). To investigate the effects of C1INH on C1s (97), a synthetic chromogenic tripeptide-p-nitroanilide substrate S-2314 (DTNB) was utilized to determine the level of active C1s. However, the substrate can also be cleaved by activated C1r and other enzymes, such as granzyme H, which prevented its use in clinic for measuring active C1s in blood (95). Thus, it is conceivable that the ideal novel assay should be highly specific and convenient by combining the specific antibody, catalytic and C2/C4 binding specificity of C1s and making use of labeling technologies, such as the fluorescence labeling.

**Table 2:** Methodology, advantages, and disadvantages of complement C1s detection.

Detection content	Methods	Clinical blood, body fluid or other samples	Quantitative or not	Advantages and disadvantages
C1s gene	PCR	Adaptive	Used to detect gene mutation and cannot be quantified	Low-flux
	NGS	Adaptive	Used to detect gene mutation and cannot be quantified	High flux, but special equipment is required
C1s protein concentration	ELISA	Adaptive	Quantitative	Low sensitivity
	Bilateral diffusion (92)	Adaptive	Semiquantitative	Low sensitivity
	Gelatin zymography (45)	Not adaptive	Semiquantitative	Operation complex
	LC-MS/MS (93)	Adaptive	Quantitative	Special equipment is required
C1s activation	Cut C2、C4 active test (94)	Not adaptive	Semiquantitative	Operation complex MASP1 and MASP2 interference
	DTNB chromogenic substrate (95)	Not adaptive, Only suitable for quantitative analysis of recombinant expression	Quantitative	Interference of granzyme H and other proteases
	S-2314 chromogenic substrate (97)	Not adaptive	Quantitative	C1r and other protease interference

## Conclusions and future directions

The critical roles of the complement system in innate and acquired immunity make the activation of C1s an informative biomarker for a variety of diseases, particularly the inflammatory, autoimmune diseases and cancers. With the success of specific antibody preparation, C1s has been validated as an effective target of therapeutic intervention. It is conceivable that more small molecular, peptide/protein, and antibodies targeting C1s for the treatment of caner, autoimmune, and infectious diseases will be developed. Thus, the complement component is a unique target for both diagnostic and therapeutic actions. Further, ongoing clinical trials and the emerging of novel therapeutics against C1s will likely bring new and better drugs into clinic in the not-so-distant future.

It has been shown recently that the expression of C1s and another two other genes is associated with Age-related macular degeneration (AMD), a progressive neurodegenerative disease of the central retina and a leading cause of vision loss in older adults worldwide (98). Mechanistically, it is likely due to their correlation with NK cell infiltration, CD4 memory T cell activation, and macrophage polarization in AMD. Thus, the expression level of the C1s gene might be utilized as a prognostic biomarker for early diagnosis and treatment of AMD. While measuring the level of C1s molecule and its activation states in clinical specimen are of great value, it remains a significant challenge to develop convenient, specific, and sensitive assays to fulfill the need, particularly determining the the kinetics of activated C1s and C1s-mediated enzymatic reaction in the serum/plasma. It is conceivable that the successful and practical clinical evaluation of C1s will enable wide applications of the biomarker in pathogenesis, diagnosis, prognosis of diseases, and for individualized therapies targeting C1s.

## Author contributions

JY and SX designed the project, JY wrote and SX revised the manuscript, PY and YY participated in the project and revised the manuscript. All authors contributed to the article and approved the submitted version.

## Funding

This work was supported by the Medical Innovation Team Foundation of Taizhou People’s Hospital (No. CXTDB201904), Research fund of the “333 project” of Jiangsu Province (No. BRA2020190), 2020 Taizhou People’s Hospital Mandatory Project (No. ZL202022) and the National Natural Science Foundation of China (No. 81871234; No. 82172763).

## Acknowledgments

We apologize for those colleagues whose important works were not cited due to limited space.

## Conflict of interest

The authors declare that the research was conducted in the absence of any commercial or financial relationships that could be construed as a potential conflict of interest.

## Publisher’s note

All claims expressed in this article are solely those of the authors and do not necessarily represent those of their affiliated organizations, or those of the publisher, the editors and the reviewers. Any product that may be evaluated in this article, or claim that may be made by its manufacturer, is not guaranteed or endorsed by the publisher.

## References

[1] Campbell CM Kahwash R. Will complement inhibition be the new target in treating COVID-19-Related systemic thrombosis? Circulation (2020) 22:1739–41. doi: 10.1161/CIRCULATIONAHA.120.047419 32271624

[2] Risitano AM Mastellos DC Huber-Lang M Yancopoulou D Garlanda C Ciceri F. Complement as a target in COVID-19? Nat Rev Immunol (2020) 20:343–4. doi: 10.1038/s41577-020-0320-7 PMC7187144 32327719

[3] Manderson AP Botto M Walport MJ. The role of complement in the development of systemic lupus erythematosus. Annu Rev Immunol (2004) 22:431–56. doi: 10.1146/annurev.immunol.22.012703.104549 15032584

[4] Berentsen S. Complement activation and inhibition in autoimmune hemolytic anemia: Focus on cold agglutinin disease. Semin Hematol (2018) 55:141–9. doi: 10.1053/j.seminhematol.2018.04.002 30032751

[5] Chimenti MS Ballanti E Triggianese P Perricone R. Vasculitides and the complement system: a comprehensive review. Clin Rev Allergy Immunol (2015) 49:333–46. doi: 10.1007/s12016-014-8453-8 25312590

[6] Skattum L van Deuren M van der Poll T Truedsson L. Complement deficiency states and associated infections. Mol Immunol (2011) 48:1643–55. doi: 10.1016/j.molimm.2011.05.001 21624663

[7] Grumach AS Kirschfink M. Are complement deficiencies really rare? overview on prevalence, clinical importance and modern diagnostic approach. Mol Immunol (2014) 61:110–7. doi: 10.1016/j.molimm.2014.06.030 25037634

[8] Frazer-Abel A Sepiashvili L Mbughuni MM Willrich MA. Overview of laboratory testing and clinical presentations of complement deficiencies and dysregulation. Adv Clin Chem (2016) 77:1–75. doi: 10.1016/bs.acc.2016.06.001 27717414

[9] Gaboriaud C Ling WL Thielens NM Bally I Rossi V. Deciphering the fine details of c1 assembly and activation mechanisms: "mission impossible"? Front Immunol (2014) 5:565. doi: 10.3389/fimmu.2014.00565 25414705 PMC4222235

[10] Wallis R Mitchell DA Schmid R Schwaeble WJ Keeble AH. Paths reunited: Initiation of the classical and lectin pathways of complement activation. Immunobiology (2010) 215:1–11. doi: 10.1016/j.imbio.2009.08.006 19783065 PMC2824237

[11] Jager U D'Sa S Schorgenhofer C Bartko J Derhaschnig U Sillaber C. Inhibition of complement C1s improves severe hemolytic anemia in cold agglutinin disease: a first-in-human trial. Blood (2019) 133:893–901. doi: 10.1182/blood-2018-06-856930 30559259 PMC6396179

[12] Roth A Barcellini W D'Sa S Miyakawa Y Broome CM Michel M. Sutimlimab in cold agglutinin disease. N Engl J Med (2021) 384:1323–34. doi: 10.1056/NEJMoa2027760 33826820

[13] Nikitin PA Rose EL Byun TS Parry GC Panicker S. C1s inhibition by BIVV009 (Sutimlimab) prevents complement-enhanced activation of autoimmune human b cells *In vitro* . J Immunol (2019) 202:1200–9. doi: 10.4049/jimmunol.1800998 PMC6360260 30635392

[14] Peerschke EI Panicker S Bussel J. Classical complement pathway activation in immune thrombocytopenia purpura: inhibition by a novel C1s inhibitor. Br J Haematol (2016) 173:942–5. doi: 10.1111/bjh.13648 PMC4973859 26305671

[15] Tosi M Duponchel C Meo T Julier C. Complete cDNA sequence of human complement cls and close physical linkage of the homologous genes cls and clr. Biochemistry-US (1987) 26:8516–24. doi: 10.1021/bi00400a004 2831944

[16] Nguyen VC Tosi M Gross MS Cohen-Haguenauer O Jegou-Foubert C de Tand MF. Assignment of the complement serine protease genes C1r and C1s to chromosome 12 region 12p13. Hum Genet (1988) 78:363–8. doi: 10.1007/BF00291737 2834284

[17] Eriksson H Nissen MH. Proteolysis of the heavy chain of major histocompatibility complex class I antigens by complement component C1s. Biochim Biophys Acta (1990) 1037:209–15. doi: 10.1016/0167-4838(90)90169-G 1689590

[18] Nissen MH Roepstorff P Thim L Dunbar B Fothergill JE. Limited proteolysis of beta 2-microglobulin at lys-58 by complement component C1s. Eur J Biochem (1990) 189:423–9. doi: 10.1111/j.1432-1033.1990.tb15505.x 2110898

[19] Busby WJ Nam TJ Moralez A Smith C Jennings M Clemmons DR. The complement component C1s is the protease that accounts for cleavage of insulin-like growth factor-binding protein-5 in fibroblast medium. J Biol Chem (2000) 275:37638–44. doi: 10.1074/jbc.M006107200 10982804

[20] Naito AT Sumida T Nomura S Liu ML Higo T Nakagawa A. Complement C1q activates canonical wnt signaling and promotes aging-related phenotypes. CELL (2012) 149:1298–313. doi: 10.1016/j.cell.2012.03.047 PMC3529917 22682250

[21] Stevens B Allen NJ Vazquez LE Howell GR Christopherson KS Nouri N. The classical complement cascade mediates CNS synapse elimination. Cell (2007) 131:1164–78. doi: 10.1016/j.cell.2007.10.036 18083105

[22] Chu Y Jin X Parada I Pesic A Stevens B Barres B. Enhanced synaptic connectivity and epilepsy in C1q knockout mice. Proc Natl Acad Sci U.S.A. (2010) 107:7975–80. doi: 10.1073/pnas.0913449107 PMC2867906 20375278

[23] Cai Y Teo BH Yeo JG Lu J. C1q protein binds to the apoptotic nucleolus and causes C1 protease degradation of nucleolar proteins. J Biol Chem (2015) 290:22570–80. doi: 10.1074/jbc.M115.670661 PMC4566231 26231209

[24] Yeo JG Leong J Arkachaisri T Cai Y Teo BH Tan JH. Proteolytic inactivation of nuclear alarmin high-mobility group box 1 by complement protease C1s during apoptosis. Cell Death Discovery (2016) 2:16069. doi: 10.1038/cddiscovery.2016.69 27648302 PMC5018544

[25] Uchio-Yamada K Tanaka M Manabe N. C1r/C1s deficiency is insufficient to induce murine systemic lupus erythematosus. Genes Immun (2019) 20:121–30. doi: 10.1038/s41435-018-0020-5 29550838

[26] Amano MT Ferriani VP Florido MP Reis ES Delcolli MI Azzolini AE. Genetic analysis of complement C1s deficiency associated with systemic lupus erythematosus highlights alternative splicing of normal C1s gene. Mol Immunol (2008) 45:1693–702. doi: 10.1016/j.molimm.2007.09.034 18062908

[27] Dragon-Durey MA Quartier P Fremeaux-Bacchi V Blouin J de Barace C Prieur AM. Molecular basis of a selective C1s deficiency associated with early onset multiple autoimmune diseases. J Immunol (2001) 166:7612–6. doi: 10.4049/jimmunol.166.12.7612 11390518

[28] Inoue N Saito T Masuda R Suzuki Y Ohtomi M Sakiyama H. Selective complement C1s deficiency caused by homozygous four-base deletion in the C1s gene. Hum Genet (1998) 103:415–8. doi: 10.1007/s004390050843 9856483

[29] Endo Y Kanno K Takahashi M Yamaguchi K Kohno Y Fujita T. Molecular basis of human complement C1s deficiency. J Immunol (1999) 162:2180–3. 9973493

[30] Abe K Endo Y Nakazawa N Kanno K Okubo M Hoshino T. Unique phenotypes of C1s deficiency and abnormality caused by two compound heterozygosities in a Japanese family. J Immunol (2009) 182:1681–8. doi: 10.4049/jimmunol.182.3.1681 19155518

[31] Kapferer-Seebacher I Pepin M Werner R Aitman TJ Nordgren A Stoiber H. Periodontal ehlers-danlos syndrome is caused by mutations in C1R and C1S, which encode subcomponents C1r and C1s of complement. Am J Hum Genet (2016) 99:1005–14. doi: 10.1016/j.ajhg.2016.08.019 PMC5097948 27745832

[32] El CS Legrand A Stoetzel C Geoffroy V Billon C Adham S. Periodontal (formerly type VIII) ehlers-danlos syndrome: Description of 13 novel cases and expansion of the clinical phenotype. Clin Genet (2021) 100:206–12. doi: 10.1111/cge.13972 33890303

[33] Bally I Dalonneau F Chouquet A Grobner R Amberger A Kapferer-Seebacher I. Two different missense C1S mutations, associated to periodontal ehlers-danlos syndrome, lead to identical molecular outcomes. Front Immunol (2019) 10:2962. doi: 10.3389/fimmu.2019.02962 31921203 PMC6930149

[34] Almitairi J Venkatraman GU Furze CM Simpson-Gray X Badakshi F Marshall JE. Structure of the C1r-C1s interaction of the C1 complex of complement activation. Proc Natl Acad Sci U.S.A. (2018) 115:768–73. doi: 10.1073/pnas.1718709115 PMC5789954 29311313

[35] Ozen A Comrie WA Ardy RC Dominguez CC Dalgic B Beser OF. CD55 deficiency, early-onset protein-losing enteropathy, and thrombosis. N Engl J Med (2017) 377:52–61. doi: 10.1056/NEJMoa1615887 28657829 PMC6690356

[36] Berentsen S Malecka A Randen U Tjonnfjord GE. Cold agglutinin disease: where do we stand, and where are we going? Clin Adv Hematol Oncol (2020) 18:35–44. 32511221

[37] Gabbard AP Booth GS. Cold agglutinin disease. Clin Hematol Int (2020) 2:95–100. doi: 10.2991/chi.k.200706.001 34595449 PMC8432332

[38] Berentsen S Hill A Hill QA Tvedt T Michel M. Novel insights into the treatment of complement-mediated hemolytic anemias. Ther Adv Hematol (2019) 10:153165097. doi: 10.1177/2040620719873321 PMC6734604 31523413

[39] Asavapanumas N Tradtrantip L Verkman AS. Targeting the complement system in neuromyelitis optica spectrum disorder. Expert Opin Biol Ther (2021) 21:1073–86. doi: 10.1080/14712598.2021.1884223 PMC8316261 33513036

[40] Pittock SJ Zekeridou A Weinshenker BG. Hope for patients with neuromyelitis optica spectrum disorders - from mechanisms to trials. Nat Rev Neurol (2021) 17:759–73. doi: 10.1038/s41582-021-00568-8 34711906

[41] Nakagawa K Sakiyama H Tsuchida T Yamaguchi K Toyoguchi T Masuda R. Complement C1s activation in degenerating articular cartilage of rheumatoid arthritis patients: immunohistochemical studies with an active form specific antibody. Ann Rheum Dis (1999) 58:175–81. doi: 10.1136/ard.58.3.175 PMC1752845 10364916

[42] Pickering RJ Naff GB Stroud RM Good RA Gewurz H. Deficiency of C1r in human serum. effects on the structure and function of macromolecular C1. J Exp Med (1970) 131:803–15. doi: 10.1084/jem.131.4.803 PMC2138784 4988128

[43] Moncada B Day NK Good RA Windhorst DB. Lupus-erythematosus-like syndrome with a familial defect of complement. N Engl J Med (1972) 286:689–93. doi: 10.1056/NEJM197203302861304 4110615

[44] Day NK Geiger H Stroud R DeBracco M Mancaido B Windhorst D. C1r deficiency: an inborn error associated with cutaneous and renal disease. J Clin Invest (1972) 51:1102–8. doi: 10.1172/JCI106902 PMC292239 4623164

[45] Ugarte-Berzal E Martens E Boon L Vandooren J Blockmans D Proost P. EDTA/gelatin zymography method to identify C1s versus activated MMP-9 in plasma and immune complexes of patients with systemic lupus erythematosus. J Cell Mol Med (2019) 23:576–85. doi: 10.1111/jcmm.13962 PMC6307758 30358100

[46] He S Lin YL. *In vitro* stimulation of C1s proteolytic activities by C1s-presenting autoantibodies from patients with systemic lupus erythematosus. J Immunol (1998) 160:4641–7. 9574573

[47] Radanova M Vasilev V Mihaylova G Kosturkova M Kishore U Roumenina L. Autoantibodies against complement classical pathway components C1q, C1r, C1s and C1-inh in patients with lupus nephritis. Int J Mol Sci (2022) 23:9281. doi: 10.3390/ijms23169281 36012546 PMC9409282

[48] Kleer JS Rabatscher PA Weiss J Leonardi J Vogt SB Kieninger-Grafitsch A. Epitope-specific anti-C1q autoantibodies in systemic lupus erythematosus. Front Immunol (2021) 12:761395. doi: 10.3389/fimmu.2021.761395 35087514 PMC8788646

[49] Csorba K Schirmbeck LA Tuncer E Ribi C Roux-Lombard P Chizzolini C. Anti-C1q antibodies as occurring in systemic lupus erythematosus could be induced by an Epstein-Barr virus-derived antigenic site. Front Immunol (2019) 10:2619. doi: 10.3389/fimmu.2019.02619 31787984 PMC6853867

[50] Kim MK Breitbach CJ Moon A Heo J Lee YK Cho M. Oncolytic and immunotherapeutic vaccinia induces antibody-mediated complement-dependent cancer cell lysis in humans. Sci Transl Med (2013) 5:163r–85r. doi: 10.1126/scitranslmed.3005361 23677592

[51] Riihila P Viiklepp K Nissinen L Farshchian M Kallajoki M Kivisaari A. Tumour-cell-derived complement components C1r and C1s promote growth of cutaneous squamous cell carcinoma. Br J Dermatol (2020) 182:658–70. doi: 10.1111/bjd.18095 PMC7065064 31049937

[52] Liu L Du X Fang J Zhao J Guo Y Zhao Y. Development of an interferon gamma response-related signature for prediction of survival in clear cell renal cell carcinoma. J Inflammation Res (2021) 14:4969–85. doi: 10.2147/JIR.S334041 PMC8485924 34611422

[53] Daugan MV Revel M Russick J Dragon-Durey MA Gaboriaud C Robe-Rybkine T. Complement C1s and C4d as prognostic biomarkers in renal cancer: Emergence of noncanonical functions of C1s. Cancer Immunol Res (2021) 9:891–908. doi: 10.1158/2326-6066.CIR-20-0532 34039653

[54] Ghebrehiwet B Ji Y Valentino A Pednekar L Ramadass M Habiel D. Soluble gC1qR is an autocrine signal that induces B1R expression on endothelial cells. J Immunol (2014) 192:377–84. doi: 10.4049/jimmunol.1302031 PMC3876932 24319267

[55] Roumenina LT Daugan MV Noe R Petitprez F Vano YA Sanchez-Salas R. Tumor cells hijack macrophage-produced complement C1q to promote tumor growth. Cancer Immunol Res (2019) 7:1091–105. doi: 10.1158/2326-6066.CIR-18-0891 31164356

[56] Heesterbeek D Angelier ML Harrison RA Rooijakkers S. Complement and bacterial infections: From molecular mechanisms to therapeutic applications. J Innate Immun (2018) 10:455–64. doi: 10.1159/000491439 PMC6784045 30149378

[57] Syed I Wooten RM. Interactions between pathogenic burkholderia and the complement system: A review of potential immune evasion mechanisms. Front Cell Infect Microbiol (2021) 11:701362. doi: 10.3389/fcimb.2021.701362 34660335 PMC8515183

[58] Johnson U Kamme C Laurell AB Nilsson NI. C1 subcomponents in acute pneumococcal otitis media in children. Acta Pathol Microbiol Scand C (1977) 85:10–6. doi: 10.1111/j.1699-0463.1977.tb03604.x 14475

[59] Messner CB Demichev V Wendisch D Michalick L White M Freiwald A. Ultra-High-Throughput clinical proteomics reveals classifiers of COVID-19 infection. Cell Syst (2020) 11:11–24. doi: 10.1016/j.cels.2020.05.012 32619549 PMC7264033

[60] Keshavarz F Ghalamfarsa F Javdansirat S Hasanzadeh S Azizi A Sabz G. Patients with covid 19 have significantly reduced CH50 activity. Virusdisease (2021) 32:1–9. doi: 10.1007/s13337-021-00710-6 34631971 PMC8486960

[61] Jarlhelt I Nielsen SK Jahn CXH Hansen CB Perez-Alos L Rosbjerg A. SARS-CoV-2 antibodies mediate complement and cellular driven inflammation. Front Immunol (2020) 12:767981. doi: 10.3389/fimmu.2021.767981 PMC8596567 34804055

[62] Lamerton RE Marcial-Juarez E Faustini SE Perez-Toledo M Goodall M Jossi SE. SARS-CoV-2 spike- and nucleoprotein-specific antibodies induced after vaccination or infection promote classical complement activation. Front Immunol (2022) 13:838780. doi: 10.3389/fimmu.2022.838780 35860286 PMC9289266

[63] Urwyler P Moser S Charitos P Heijnen I Rudin M Sommer G. Treatment of COVID-19 with conestat Alfa, a regulator of the complement, contact activation and kallikrein-kinin system. Front Immunol (2020) 11:2072. doi: 10.3389/fimmu.2020.02072 32922409 PMC7456998

[64] Sharp JA Whitley PH Cunnion KM Krishna NK. Peptide inhibitor of complement c1, a novel suppressor of classical pathway activation: mechanistic studies and clinical potential. Front Immunol (2014) 5:406. doi: 10.3389/fimmu.2014.00406 25202312 PMC4141160

[65] Travins JM Ali F Huang H Ballentine SK Khalil E Hufnagel HR. Biphenylsulfonyl-thiophene-carboxamidine inhibitors of the complement component C1s. Bioorg Med Chem Lett (2008) 18:1603–6. doi: 10.1016/j.bmcl.2008.01.064 18242991

[66] Chen JJ Schmucker LN Visco DP. Pharmaceutical machine learning: Virtual high-throughput screens identifying promising and economical small molecule inhibitors of complement factor C1s. Biomolecules (2018) 8:24. doi: 10.3390/biom8020024 PMC6023033 29735903

[67] Szilagyi K Hajdu I Flachner B Lorincz Z Balczer J Gal P. Design and selection of novel C1s inhibitors by *in silico* and *in vitro* approaches. Molecules (2019) 24:3641. doi: 10.3390/molecules24203641 PMC6832932 31600984

[68] Karnaukhova E. C1-inhibitor: Structure, functional diversity and therapeutic development. Curr Med Chem (2022) 29:467–88. doi: 10.2174/0929867328666210804085636 34348603

[69] Davis AR. Biological effects of C1 inhibitor. Drug News Perspect (2004) 17:439–46. doi: 10.1358/dnp.2004.17.7.863703 15514703

[70] Lunn M Santos C Craig T. Cinryze as the first approved C1 inhibitor in the USA for the treatment of hereditary angioedema: approval, efficacy and safety. J Blood Med (2010) 1:163–70. doi: 10.2147/JBM.S9576 PMC3262319 22282695

[71] US National Library Of Medicine. ClinicalTrials.gov (2021). Available at: https://www.clinicaltrials.gov/show/NCT02502903.

[72] US National Library Of Medicine. ClinicalTrials.gov (2021). Available at: https://www.clinicaltrials.gov/show/NCT03347422.

[73] US National Library Of Medicine. ClinicalTrials.gov (2021). Available at: https://www.clinicaltrials.gov/show/NCT03347396.

[74] US National Library Of Medicine. ClinicalTrials.gov (2021). Available at: https://www.clinicaltrials.gov/show/NCT03275454.

[75] US National Library Of Medicine. ClinicalTrials.gov (2021). Available at: https://www.clinicaltrials.gov/show/NCT04669600.

[76] US National Library Of Medicine. ClinicalTrials.gov (2021). Available at: https://www.clinicaltrials.gov/show/NCT04802057.

[77] US National Library Of Medicine. ClinicalTrials.gov (2021). Available at: https://www.clinicaltrials.gov/show/NCT04269551.

[78] US National Library Of Medicine. ClinicalTrials.gov (2021). Available at: https://www.clinicaltrials.gov/show/NCT04658472.

[79] Gavriilaki E de Latour RP Risitano AM. Advancing therapeutic complement inhibition in hematologic diseases: PNH and beyond. Blood (2022) 139:3571–82. doi: 10.1182/blood.2021012860 34482398

[80] Kushner CJ Payne AS. Increasing the complement of therapeutic options in bullous pemphigoid. J Invest Dermatol (2018) 138:246–8. doi: 10.1016/j.jid.2017.09.026 29389324

[81] Kasprick A Holtsche MM Rose EL Hussain S Schmidt E Petersen F. The anti-C1s antibody TNT003 prevents complement activation in the skin induced by bullous pemphigoid autoantibodies. J Invest Dermatol (2018) 138:458–61. doi: 10.1016/j.jid.2017.08.030 28899686

[82] Thomas KA Valenzuela NM Gjertson D Mulder A Fishbein MC Parry GC. An anti-C1s monoclonal, TNT003, inhibits complement activation induced by antibodies against HLA. Am J Transplant (2015) 15:2037–49. doi: 10.1111/ajt.13273 PMC4654252 25904443

[83] Valenzuela NM Thomas KA Mulder A Parry GC Panicker S Reed EF. Complement-mediated enhancement of monocyte adhesion to endothelial cells by HLA antibodies, and blockade by a specific inhibitor of the classical complement cascade, TNT003. Transplantation (2017) 101:1559–72. doi: 10.1097/TP.0000000000001486 PMC5482566 28640789

[84] Muhlbacher J Jilma B Wahrmann M Bartko J Eskandary F Schorgenhofer C. Blockade of HLA antibody-triggered classical complement activation in sera from subjects dosed with the anti-C1s monoclonal antibody TNT009-results from a randomized first-in-Human phase 1 trial. Transplantation (2017) 101:2410–8. doi: 10.1097/TP.0000000000001804 PMC5610566 28926521

[85] Lewis LA Panicker S DeOliveira RB Parry GC Ram S. Effect of a C1s inhibitor on the efficacy of anti-capsular antibodies against neisseria meningitidis and streptococcus pneumoniae. Immunohorizons (2019) 3:519–30. doi: 10.4049/immunohorizons.1900031 31690560

[86] Dhillon S. Sutimlimab: First approval. Drugs (2022) 82:817–23. doi: 10.1007/s40265-022-01711-5 35412113

[87] Roos A Wieslander J. Evaluation of complement function by ELISA. Methods Mol Biol (2014) 1100:11–23. doi: 10.1007/978-1-62703-724-2_2 24218247

[88] Seelen MA Roos A Wieslander J Mollnes TE Sjoholm AG Wurzner R. Functional analysis of the classical, alternative, and MBL pathways of the complement system: standardization and validation of a simple ELISA. J Immunol Methods (2005) 296:187–98. doi: 10.1016/j.jim.2004.11.016 15680163

[89] Roumenina LT Sene D Radanova M Blouin J Halbwachs-Mecarelli L Dragon-Durey MA. Functional complement C1q abnormality leads to impaired immune complexes and apoptotic cell clearance. J Immunol (2011) 187:4369–73. doi: 10.4049/jimmunol.1101749 21930969

[90] Mollnes TE Jokiranta TS Truedsson L Nilsson B Rodriguez DCS Kirschfink M. Complement analysis in the 21st century. Mol Immunol (2007) 44:3838–49. doi: 10.1016/j.molimm.2007.06.150 17768101

[91] Dasdemir S Yildiz M Celebi D Sahin S Aliyeva N Haslak F. Genetic screening of early-onset patients with systemic lupus erythematosus by a targeted next-generation sequencing gene panel. Lupus (2022) 31:330–7. doi: 10.1177/09612033221076733 35086391

[92] Ziccardi RJ Cooper NR. Demonstration and quantitation of activation of the first component of complement in human serum. J Exp Med (1978) 147:385–95. doi: 10.1084/jem.147.2.385 PMC2184484 75238

[93] Kodeboyina SK Lee TJ Bollinger K Ulrich L Bogorad D Estes A. Aqueous humor proteomic alterations associated with visual field index parameters in glaucoma patients: A pilot study. J Clin Med (2021) 10:1180. doi: 10.3390/jcm10061180 33808966 PMC8001447

[94] Matsumoto M Nagaki K Kitamura H Kuramitsu S Nagasawa S Seya T. Probing the C4-binding site on C1s with monoclonal antibodies. evidence for a C4/C4b-binding site on the gamma-domain. J Immunol (1989) 142:2743–50. 2522968

[95] Edwards KM Kam CM Powers JC Trapani JA. The human cytotoxic T cell granule serine protease granzyme h has chymotrypsin-like (chymase) activity and is taken up into cytoplasmic vesicles reminiscent of granzyme b-containing endosomes. J Biol Chem (1999) 274:30468–73. doi: 10.1074/jbc.274.43.30468 10521426

[96] Nakagawa K Sakiyama H Tsuchida T Yamaguchi K Toyoguchi T Masuda R. Complement C1s activation in degenerating articular cartilage of rheumatoid arthritis patients: immunohistochemical studies with an active form specific antibody. Ann Rheum Dis (1999) 58:175–81. doi: 10.1136/ard.58.3.175 PMC1752845 10364916

[97] Nilsson T Wiman B. Kinetics of the reaction between human C1-esterase inhibitor and C1r or C1s. Eur J Biochem (1983) 129:663–7. doi: 10.1111/j.1432-1033.1983.tb07100.x 6297891

[98] Zeng Y Yin X Chen C Xing Y. Identification of diagnostic biomarkers and their correlation with immune infiltration in age-related macular degeneration. Diagnostics (2021) 11:1079. doi: 10.3390/diagnostics11061079 34204836 PMC8231534

